# Lack of β2-AR Increases Anxiety-Like Behaviors and Rewarding Properties of Cocaine

**DOI:** 10.3389/fnbeh.2017.00049

**Published:** 2017-03-13

**Authors:** Huiwen Zhu, Zhiyuan Liu, Yiming Zhou, Xuming Yin, Bo Xu, Lan Ma, Xing Liu

**Affiliations:** State Key Laboratory of Medical Neurobiology, School of Basic Medical Sciences and the Institutes of Brain Science, Collaborative Innovation Center for Brain Science, Fudan UniversityShanghai, China

**Keywords:** β2-AR, anxiety, depression, reward, cocaine

## Abstract

It is well known that β-adrenoceptors (β-ARs) play a critical role in emotional arousal and stressful events, but the specific contributions of the β2-AR subtype to the psychological disorders are largely unknown. To investigate whether β2-AR are involved in anxiety-like behavior and reward to addictive drugs, we conducted a series of behavioral tests on β2-AR knock-out (KO) mice. β2-AR KO mice exhibited increased preference for the dark compartment and closed arm in tests of Light/Dark box and elevated plus maze, indicating that β2-AR deletion elevates level of anxiety or innate fear. β2-AR KO mice also showed decreased immobility in tail suspension test (TST), suggesting that β2-AR deletion inhibits depression-like behavior. Interestingly, β2-AR ablation did not change basal locomotion but significantly increased locomotor activity induced by acute cocaine administration. β2-AR KO mice showed enhanced place preference for cocaine, which could be attenuated by β1-selective AR antagonist betaxolol. Consistently, β2-AR agonist suppressed cocaine-conditioned place preference (CPP). These data indicate that β2-AR deletion enhances acute response and reward to cocaine. Our results suggest that β2-AR regulates anxiety level, depression-like behavior and hedonic properties of cocaine, implicating that β2-AR are the potential targets for the treatment of emotional disorders and cocaine addiction.

## Introduction

Anxiety and depression are the common types of psychiatric disorders, with high incidence and life-long prevalence (Kessler et al., 2005; Mufaddel et al., 2013). Psychological stressors activate neuroendocrine pathways to release catecholamines, and β-adrenoceptors (β-ARs) play a critical role in emotional arousal and stressful events (Blanchard et al., 2001). β-ARs, consisting of β1, β2 and β3 subtypes, are prototypical members of G protein-coupled receptor (GPCR) family. The β1-AR and β2-AR are abundant in the brain and their functions in the neural system are studied. Human studies showed that the density of cortical β-ARs were lower among antidepressant-free depressed suicide victims (De Paermentier et al., 1989). Increased noradrenaline levels in brain lead to improved emotional memory performance and β-adrenergic antagonists applied in comparable time-frames lead to reduced memory performance on stress tasks (McGaugh and Introini-Collison, 1987; Cahill et al., 1994; Cahill and Alkire, 2003). Propranolol, a non-selective β-AR antagonist with equal affinity for both β1- and β2-AR (Rehsia and Dhalla, 2010), was studied as a general anxiolytic treatment of anxiety disorders, PTSD and addiction for its amnesic effect (Famularo et al., 1988; Steenen et al., 2016). Animal studies showed that stress-induced anxiety-like behavior and neuronal activation were prevented by propranolol (Wohleb et al., 2011). Considering many medications non-selectively modulate the adrenergic system and β1- and β2-AR may be dissociated functionally, understanding the role of subtypes of β-ARs in emotional behaviors is important and has significant clinical implications.

Activation of either subtype of β-ARs results in stimulation of adenylyl cyclase (Levy et al., 1993), but differences in receptor distribution and efficacy have been found. β1-AR are in much higher levels than β2-AR within forebrain structures such as the cerebral cortex, caudate, hippocampus and amygdala (Ordway et al., 1988). While isoproterenol, which has equal affinity for both β1 and β2 subtypes, induces greater adenylyl cyclase activity upon stimulation of β2-AR than β1-AR (Green et al., 1992). Up to date, the functions of these subtypes of β-ARs in brain are not very well distinguished. Especially, the role of β2-AR in the regulation of emotional behaviors is to be clarified.

Recently, studies focused on the functions of β2-AR in central nervous system have revealed that prefrontal cortical β2-AR activate spike-timing-dependent LTP and enhances fear memory by stimulating postsynaptic cAMP-PKA signaling and suppressing GABAergic circuit activities (Zhou et al., 2013). Astrocytic rather than neuronal β2-AR in the hippocampus plays a key role in the consolidation of contextual fear memory (Gao et al., 2016). Methyl-CpG-binding protein 2 (MeCP2) gene is an X-linked gene encoding the MeCP2 protein. Mutation of this gene may cause neurodevelopmental disorders, such as Rett syndrome (RTT) and decreased synaptic plasticity (Kishi and Macklis, 2004; Cronk et al., 2015; Hara et al., 2015; Lombardi et al., 2015; Tai et al., 2016). β2-AR agonist clenbuterol rescued deficits in social memory in young male Mecp2-null and improved memory performance in object recognition and decreased anxiety in heterozygous (HET) female mice (Mellios et al., 2014). β2-AR activation stimulates CRF-releasing neurons in the BNST that interface with motivational neural circuitry to induce reinstatement of cocaine conditioned reward (McReynolds et al., 2014). However, the roles of β2-AR in psychiatric disorders are largely unknown.

In this study, we investigated the potential roles of β2-AR in regulating emotional behaviors. We showed that β2-AR knock-out (KO) resulted in increased anxiety in the light-dark box and elevated plus maze tasks, decreased depression-like behavior in tail-suspension test (TST), and elevated cocaine-induced rewarding effects in locomotion and conditioned placed preference tasks. Our data suggest the necessity of β2-AR in control of anxiety and response to cocaine.

## Materials and Methods

### Animals

**Figure 1:**
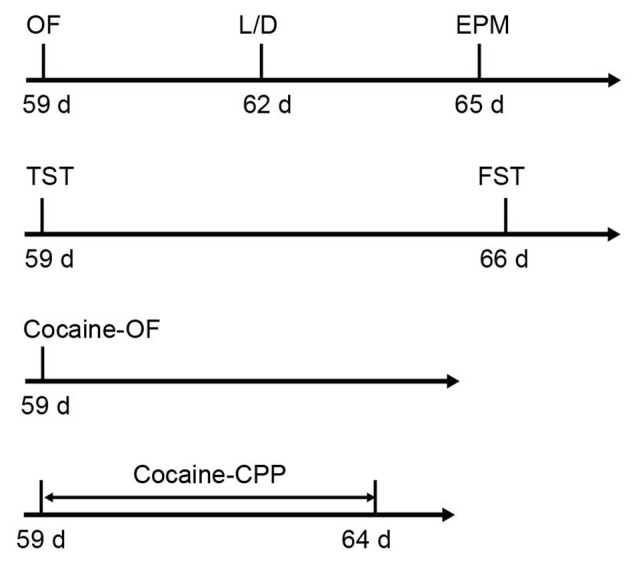
**Sequence of behavioral tests.** The anxiety level and depression level tests were carried out following the first and second cohorts, respectively. Acute cocaine response and cocaine conditioned place preference (CPP) were tested following the third and fourth cohorts, respectively (the first cohort: wild type (WT) *n* = 26–27, heterozygous (HET) *n* = 17, knock-out (KO) *n* = 27; the second cohort: WT *n* = 12, HET *n* = 18, KO *n* = 16; the third cohort: WT *n* = 9, HET *n* = 6, KO *n* = 9; the forth cohort: WT *n* = 18, HET *n* = 8, KO *n* = 20).

Six-week-old male C57BL/6J mice were purchased form Slaccas Lab Animal Ltd, Shanghai, China, weighing about 22 g. β2-AR KO mice were the gift from Prof. Gang Pei (SIBS, Chinese Academy of Sciences) and backcrossed onto a C57BL/6J background. Experiments were carried out in wild type (WT), HET and KO littermates. All animals were housed with a reversed 12 h light/dark cycle and with access to food and water available *ad libitum*. Treatments were strictly in accordance with the National Institutes of Health Guide for the Care and Use of Laboratory Animals and were approved by Animal Care and Use Committee of Shanghai Medical College of Fudan University. The male mice with 8–10 weeks’ age were used for all behavioral tests. The behavioral tests were performed in the following orders: open field (OF), light/dark box and elevated plus maze tasks were carried out with the first cohort of mice (WT: *n* = 26–27, HET: *n* = 17, KO: *n* = 27). TST and forced swimming test (FST) were taken out with the second cohort (WT: *n* = 12, HET: *n* = 18, KO: *n* = 16). The third cohort (WT: *n* = 9, HET: *n* = 6, KO: *n* = 9) were submitted to locomotion tests induced by acute cocaine administration and the forth cohort (WT: *n* = 18, HET: *n* = 8, KO: *n* = 20) were submitted to cocaine conditioned place preference (CPP; Figure 1). β2-AR KO mice and subsequent offspring were genotyped using the following primer sets: 5′-CAC GAG ACT AGT GAG ACG TG-3′; 5′-ACC AAG AAT AAG GCC CGA GT-3′; 5′-CCG GGA ATA GAC AAA GAC CA-3′.

### Reagents

Cocaine hydrochloride (Qinghai Pharmaceutical Firm, China) was dissolved in 0.9% saline at 2 mg/ml for mouse CPP, 4 mg/ml for locomotor response test. Betaxolol (Tocris Bioscience, UK), a selective β1-AR antagonist, was dissolved in saline at 2 mg/ml and administered at a dose of 10 mg/kg (i. p.; Vranjkovic et al., 2012; Al-Hasani et al., 2013). Clenbuterol (Tocris Bioscience, UK), a selective β2-AR agonist, was dissolved in saline at 1 mg/ml and administering at a dose of 5 mg/kg (i. p.; Heal et al., 1991; Mellios et al., 2014). Control animals received an equivalent volume of saline.

### Western Blotting

Mice brains were removed on ice, hippocampus and medial prefrontal cortex (mPFC) were rapidly dissected and the tissues were prepared as following. Briefly, brain samples were homogenized in ice-cold 0.32 M sucrose, 5 mM HEPES (pH 7.4), 0.1 mM EDTA, and protease inhibitors mixture in a glass homogenizer. Homogenates were centrifuged (1000× g, 10 min, 4°C), and the supernatants were spun at 14,000× g for 30 min in a centrifuge at 4°C. Then the pellets were resuspended in 200 μL of 1× P buffer (5.4 mM KCl, 0.8 mM MgSO_4_, 5.5 mM glucose, 50 mM HEPES, 130 mM choline chloride, 1 mM BSA, and 0.01% CHAPS). The protein concentration was determined by BCA assay (Pierce, 23235). Equal amounts of total protein (30 μg) isolated from cell membrane of the hippocampus and mPFC from WT and β2-AR KO mice were loaded on 10% sodium dodecyl sulfate polyacrylamide gels and then transferred to polyvinylidene difluoride membranes (Amersham Pharmacia Biotech, Piscataway, NJ, USA). Then the membranes were incubated in primary antibody for β1-AR (1:100, Santa cruz), β2-AR (1:100, Santa cruz) or β-tubulin (1:2500; Sigma, St Louis, MO, USA) at 4°C overnight. The membranes were then incubated with corresponding secondary antibody (1:50,000, Jackson Immuno Research) for 2 h after washing in TBST for three times. Protein bands were visualized using odyssey (LI-COR Biosciences). The immunoblots were analyzed with Image-Pro Plus to measure the optical density of the bands of β1-AR and β2-AR. Data of β-AR protein levels for WT and KO mice were expressed as percentage of the averaged values of β-tubulin.

### Open Field (OF)

Three days prior to locomotor activity assessments, mice were habituated to the testing room for 30 min each day. During the test, mice were released from the center of an OF test chamber (20 × 20 cm^2^, ENV-515; Med Associates, Inc., St. Albans, VT, USA) and were allowed to explore the arena freely for 30 min. Total distance, distance and duration in the center area, entrance times to the center area were analyzed using OF Activity Software package v. 4.0 (Med Associates, Inc.). Testing was conducted with 25 Lux illuminance in the chamber. In the test for locomotor activity induced by acute cocaine administration, mice were placed in the OF chamber for 30 min followed by another 60 min after an injection of cocaine (20 mg/kg i.p.).

### Light-Dark Box (L/D)

The light/dark box (46 × 27 × 30 cm) is composed of two compartments: the dark compartment (one third of the box) and the light compartment (two thirds of the box). Testing was conducted with 25 Lux illuminance in the light box (Birkett et al., 2011; Lehmann and Herkenham, 2011; Bluett et al., 2014; Shonesy et al., 2014). Mice were placed in the center of the light box and allowed full access to both compartments for 6 min. The tasks were taped by a digital video camera and the time mice spent in each compartment was analyzed with Clever System software (CleverSys).

### Elevated Plus Maze (EPM)

Anxiety-related behavior was also measured by EPM task as previously described (Pravetoni and Wickman, 2008). Briefly, the maze was elevated 50 cm above the ground and consisted of two open arms, and two closed arms, as well as an exposed center panel. The mice were placed in the center of the maze, facing the closed arm. The sessions were taped by a digital video camera for 6 min and the time spent in each arm was analyzed with Clever System software (CleverSys). Time spent in the EPM center was not included. Testing was conducted under standard room lighting conditions.

### Tail Suspension Test (TST)

The TST as one of the most widely used models for assessing the antidepressant-like activity in mice was used in our experiment. Mice were suspended 50 cm above the floor by the tip of the tail (<2 cm) with adhesive tape from a horizontal bar and were positioned such that the base of their tails was vertical to the bottom of the bar. The tasks were taped by a digital video camera for 5 min. Immobility was analyzed with the Tail Suspension Software (CleverSys). Mice were considered immobile only when they hung passively and completely motionless.

### Forced Swimming Test (FST)

FST is another widely used model for assessing the antidepressant-like activity in mice. Mice were gently released into a transparent Plexiglas cylinder (diameter 10 cm, height 25 cm) filled with water (25 ± 0.5°C, 20 cm high) for a 5 min session (under <500 lux ambient light). Immobility (i.e., cessation of limb movement except minor involuntary movement of the hind limbs) was scored with the ForcedSwimScan software (CleverSys). Each mouse was judged to be immobile when it ceased struggling and remained floating motionless in the water, making only those movements necessary to keep its head above water as previous reported (Kaster et al., 2007).

### Cocaine Conditioned Place Preference (Cocaine-CPP)

A two-chamber, unbiased CPP paradigm was applied in accordance with the method previously employed (Liu et al., 2015). The CPP apparatus consisted of two compartments with distinct flooring and walls. Before each session mice were habituated to the experimental room for at least 30 min. On day 1, mice were allowed free access to both sides of apparatus for 15 min, and the duration in the two different chambers were recorded respectively. Mice with an initial preference (>65% of total time) for either chamber were excluded from the experiment. On day 2, mice were subjected to the conditioning phase, they were confined to one of the conditioning compartments for 30 min after injection of saline (4 ml/kg, AM) and confined to the other compartment after injection of cocaine (10 mg/kg, PM). On day 3 the training were conducted with switched order of the saline and cocaine conditioning. On day 4 the conditioning was performed as day 2. On day 5, mice were allowed free access to the entire apparatus, and the duration in each side was recorded. During the conditioning, clenbuterol or betaxolol were injected 30 min before cocaine conditioning. The sessions were taped by a digital video camera and the time spent in each chamber recorded by a trained observer blind to the genotype and treatment. The CPP preference was determined as score with time (s) spent in cocaine paired side minus saline side.

### Statistical Analysis

Experimental data were presented as the mean ± SEM and analyzed by Sigmaplot 12.5. The behavioral results from OF, L/D box, EPM, TST, FST and CPP were analyzed with one-way ANOVA or two-way repeated measures (RM) ANOVA followed by the Bonferroni’s *post hoc* test. The WB results were analyzed with two-tailed Student’s *t*-test.

## Results

### β2-AR Knock-Out Increases Level of Anxiety

**Figure 2:**
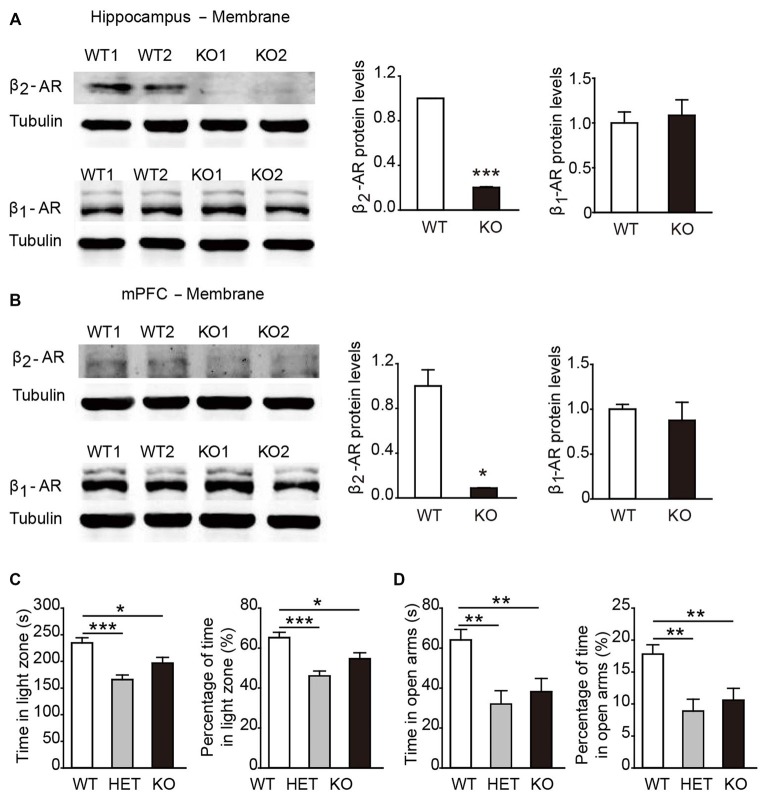
**Anxiety level was elevated in β2-adrenoceptors (β2-AR) KO mice. (A)** Representative images of β2-AR and β1-AR expression in the hippocampus of the WT and β2-AR KO mice by WB analysis and the quantification of β1-AR and β2-AR expression in hippocampus membrane fractions. **(B)** Representative images of β2-AR and β1-AR expression in the medial prefrontal cortex (mPFC) of the WT and β2-AR KO and the quantification of β1-AR and β2-AR expression in mPFC membrane fractions. **(C,D)** The anxiety level of WT, HET and β2-AR KO mice. **(C)** Light/Dark box test WT: *n* = 27, HET: *n* = 17, β2-AR KO: *n* = 27.** (D)** Elevated plus maze test. WT: *n* = 26, HET: *n* = 17, β2-AR KO: *n* = 27. Data are presented as mean ± SEM *p < 0.05 vs. WT, **p < 0.01 vs. WT, ***p < 0.001 vs. WT.

In general, β2-AR KO mice showed no genotype-dependent differences in body weight, no obvious developmental abnormalities, or deficits in touch, vision and hearing compared with their WT littermates. To verify the deletion of β2-AR, the hippocampus and mPFC were dissected from β2-AR KO mice and WT mice for Western blot analysis (Figures 2A,B). The results showed deficient expression of β2-AR and normal expression levels of β1-AR in the hippocampus and mPFC of the β2-AR KO mice (Figure 2A, β2-AR: *p* < 0.001, β1-AR: *p* = 0.732; Figure 2B, β2-AR: *p =* 0.024, β1-AR: *p* = 0.614, *t*-test).

In the L/D box and EPM tasks, both β2-AR KO and HET mice spent significantly less amounts of time in the light compartment and the open arms than WT mice (Figure 2C, *F*_(2,67)_ = 10.638, *p* < 0.001, one-way ANOVA; Figure 2D, *F*_(2,66)_ = 7.322, *p* = 0.001, one-way ANOVA). The results from both tests consistently showed that the anxious state and innate fear were increased by deletion of one or two copies of Adrb2 and sufficient β2-AR expression might have the anxiolytic effects.

### β2-AR Knock-Out Attenuates Depression-Like Behaviors

**Figure 3:**
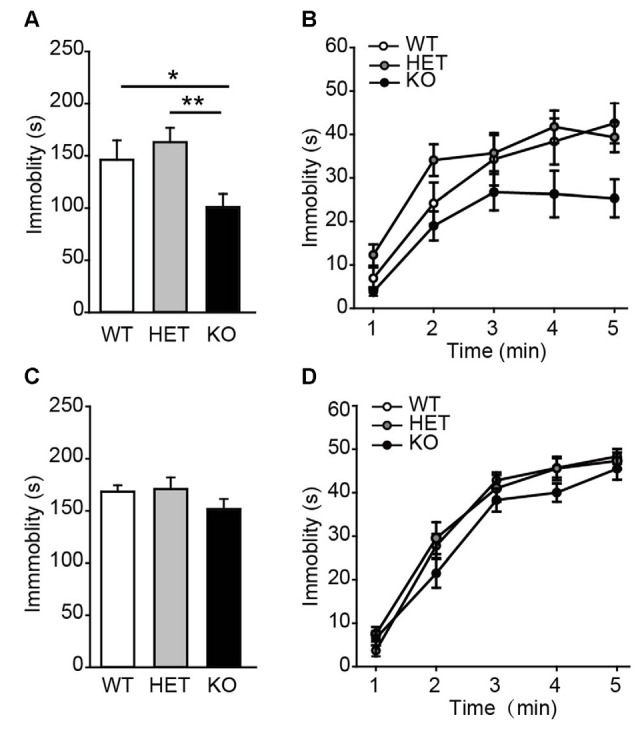
**Depression-like behavior was suppressed in β2-AR KO mice.** The performance of WT, HET and β2-AR KO mice in Tail-suspension **(A,B)** and Forced swimming **(C,D)** tests. Total immobility time during 5-min period **(A,C)** in each 1-min block **(B,D)** are plotted. For tail-suspension test (TST): WT: *n* = 12; HET: *n* = 18; KO: *n* = 16. For Forced swimming test (FST): WT: *n* = 12; HET: *n* = 18; KO: *n* = 16). Data are presented as mean ± SEM *p < 0.05 vs. WT, **p < 0.01 vs. HET.

Next, the performance of β2-AR KO mice was assessed in TST and FST, two behavioral assays widely used to examine behavioral despair in mice (Porsolt et al., 1977; Steru et al., 1985). In TST, β2-AR KO mice displayed significantly less immobility during the entire 5 min than WT and HET mice (Figure 3A, *F*_(2,43)_ = 6.315, *p* = 0.004, one-way ANOVA), however, immobility time during each 5-min were indistinguishable of the test (Figure 3B, *F*_genotype × time(2,43)_ = 1.344, *p* = 0.255, two-way RM ANOVA). In FST, immobility duration in total or each 5 min was comparable in β2-AR KO, HET and WT mice (Figure 3C, *F*_(2,43)_ = 1.137, *p* = 0.33, one-way ANOVA; Figure 3D, *F*_genotype × time(2,43)_ = 1.356, *p* = 0.219, two-way RM ANOVA). These results suggest that behavioral despair is suppressed by β2-AR ablation in TST.

### β2-AR Knock-Out Increases Response to Acute Cocaine

**Figure 4:**
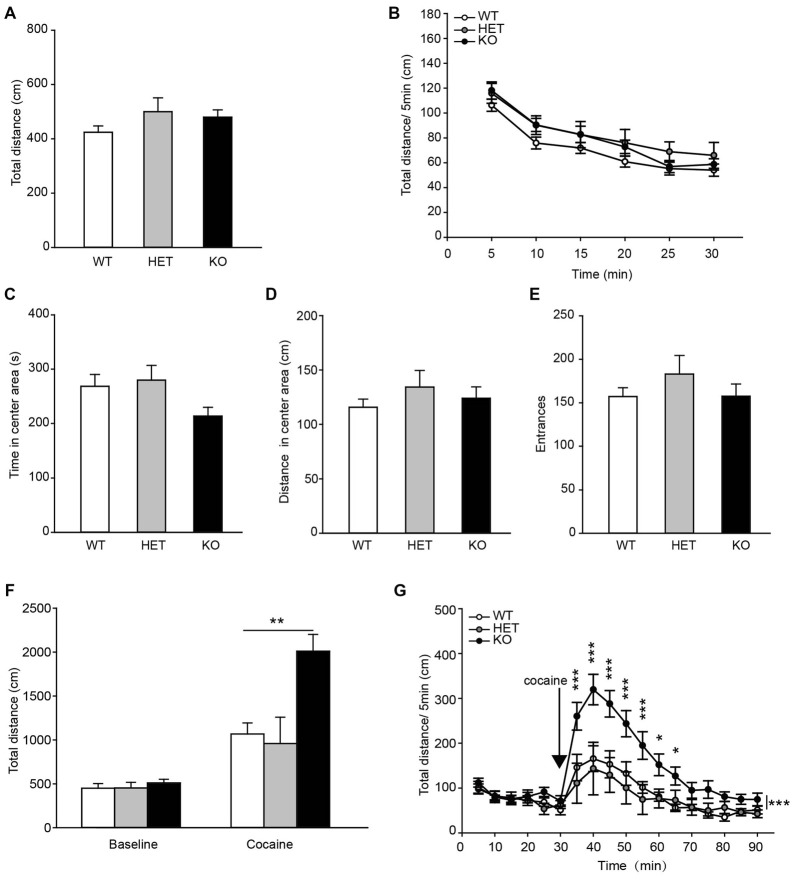
**Locomotor response to acute cocaine administration increased in β2-AR KO mice.** The performance of WT, HET and β2-AR KO mice in Open field (OF) **(A–E)**. **(A)** Total distance traveled in the whole arena for 30 min.** (B)** Distance traveled during each 5-min block in the whole arena. **(C)** Time spent and **(D)** distance traveled in the center arena (one-quarter of the total area around the center of the arena). **(E)** Number of entrances into center arena (WT: *n* = 27, HET: *n* = 17, KO: *n* = 27). Data are presented as the mean ± S.E.M. The locomotor activity induced by an injection of cocaine (20 mg/kg, i. p.) was tested after 30-min adaptation in OF in WT, HET and β2-AR KO mice **(F,G)**. **(F)** Total distance traveled in the whole arena for a 60-min duration after cocaine injection. **(G)** Distance traveled during each 5-min block in the whole arena (WT: *n* = 9, HET: *n* = 6, KO: *n* = 9). Data are presented as the mean ± SEM. *p < 0.05 vs. WT and HET, **p < 0.01 vs. WT and HET, ***p < 0.001 vs. WT and HET.

To assess the locomotor activity in β2-AR KO mice, the OF task was performed. The data showed that the total distances traveled by β2-AR KO mice, HET and WT littermates during both the entire test period and each 5-min were indistinguishable (Figure 4A, *F*_(2,68)_ = 1.506, *p* = 0.229, one-way ANOVA; Figure 4B, *F*_genotype × time(2,68)_ = 1.506, *p* = 0.229, RM two-way ANOVA). β2-AR KO mice did not show difference in time spent in the center of the chamber and distance traveled, or number of entries into the center of the OF chamber from HET and WT mice (Figures 4C–E, *F*_(2,68)_ = 2.857, *p* = 0.064; *F*_(2,68)_ = 0.680, *p* = 0.510; *F*_(2,68)_ = 0.863, *p* = 0.462; one-way ANOVA). These results indicate that β2-AR KO does not change locomotor activity.

Acute cocaine (20 mg/kg, i.p.) administration significantly increased total distance traveled in WT, HET and β2-AR KO mice, but β2-AR KO mice displayed significantly higher locomotor activity than WT and HET mice (Figure 4F, *F*_genotype × treatment(2,22)_ = 7.604, *p* = 0.003, two-way RM ANOVA) during a 60-min period following cocaine injection in the OF test. Two-way RM ANOVA revealed a significant genotype by time interaction and Bonferroni’s *post hoc* revealed that acute cocaine treatment induced an enhanced locomotor activity in β2-AR KO mice, the increase in locomotor activity primarily took place during the first 35 min of the test (Figure 4G, *F*_genotype × time(2,22)_ = 4.132, *p* < 0.001). The results indicate that ablation of β2-AR enhances response to acute cocaine administration.

### β2-AR Knock-Out Increases Cocaine-Conditioned Place Preference

**Figure 5:**
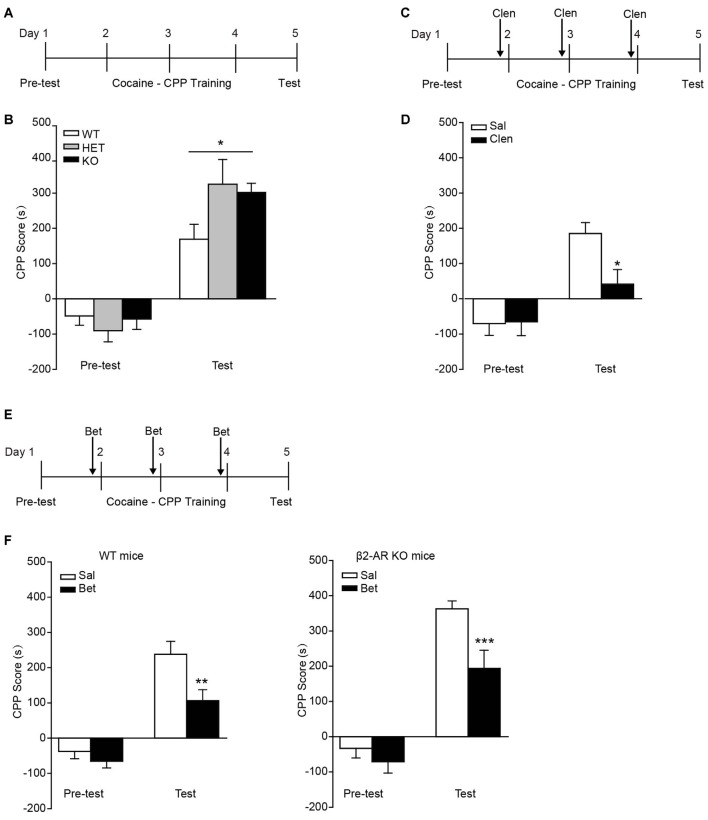
**Preference for cocaine paired side was enhanced in β2-AR KO mice.** The cocaine-CPP was conducted in WT, HET and β2-AR KO mice. After three daily conditioning, mice were tested for the preference for cocaine paired side. **(A,B)** Cocaine-CPP procedure **(A)** and the memory retention test** (B)**. WT: *n* = 18, HET: *n* = 8, KO: *n* = 20. *p < 0.05 vs. WT. **(C,D)** Cocaine-CPP procedure **(C)** and the memory retention test **(D)** for C57 mice. Clenbuterol (Clen, 5 mg/kg, i.p.) or saline was administrated 30 min before cocaine conditioning. *n* = 14–20 per group. *p < 0.05 vs. saline group. **(E,F)** Cocaine-CPP procedure **(E)** and the memory retention test **(F)** for WT (left) and β2-AR KO (right) mice. Betaxolol (Bet, 10 mg/kg, i.p.) or saline was injected 30 min before cocaine conditioning. WT/Sal: *n* = 24, WT/Bet: *n* = 18, KO/Sal: *n* = 17, KO/Bet: *n* = 14). Data are presented as the mean ± SEM. **p < 0.01 and ***p < 0.001 vs. saline group.

In the task of cocaine-CPP, all groups showed preference for cocaine-paired side under the dose of 10 mg/kg cocaine (Figure 5A). Two-way RM ANOVA revealed a significant genotype by session interaction (Figure 5B, *F*_genotype × session(2,43)_ = 3.827, *p* = 0.03), and Bonferroni’s *post hoc* revealed a significant higher preference for the cocaine-paired side developed in β2-AR KO and HET mice compared with WT mice, indicating β2-AR KO increased rewarding properties of cocaine. To further verify the role of β2-AR in cocaine rewarding effect, clenbuterol (5 mg/kg), was administrated 30 min before each cocaine conditioning session (Figure 5C). CPP scores were recorded 24 h after the last conditioning session. Clenbuterol treatment significantly reduced the preference for cocaine-paired side (Figure 5D, *F*_treatment × session(1,24)_ = 7.439, *p* = 0.012, two-way RM ANOVA). With regard to the normal expression level of β1-AR in β2-AR KO mice, the role of β1-AR in cocaine-CPP was tested. Betaxolol (10 mg/kg) was given 30 min before cocaine conditioning daily (Figure 5E). We found that betaxolol inhibited the preference for cocaine-paired side in WT and β2-AR KO mice, however, antagonism of β1-AR in β2-AR KO mice only reduced the CPP score to the levels of cocaine-CPP developed in WT mice rather than a completed disruption, further suggesting that β2-AR deletion enhanced rewarding effect of cocaine (Figure 5F left, *F*_treatment × session(1,40)_ = 4.176, *p* = 0.048, two-way RM ANOVA; Figure 5F right, *F*_treatment × session(1,29)_ = 4.667, *p* = 0.039, two-way RM ANOVA). The above results indicate that β2-AR might suppress rewarding effects induced by cocaine, producing effects opposite to the function of β1-AR.

## Discussion

To investigate the role of β2-AR in stressful or emotional behaviors, a battery of behavioral tests was performed on β2-AR KO mice. In this study, the β2-AR KO mice showed increased preference for the dark compartment and closed arm in L/D and EPM tests. β2-AR KO mice also showed decreased immobility in TST. Interestingly, β2-AR deletion did not change basal locomotor activity, but significantly increased locomotor activity induced by acute cocaine administration and greatly enhanced preference for cocaine, which was significantly attenuated by betaxolol, the selective β1-AR antagonist. Consistently, the β2-AR agonist disrupted preference for cocaine. Our results suggest that β2-AR play pivotal roles in regulating anxiety, depression-like behaviors and reward induced by cocaine.

β2-AR are widely distributed in the brain, including frontal and piriform cortices, the medial septal nuclei, the olfactory tubercle, the hippocampus and the midbrain (Asanuma et al., 1991). In this study, WB analysis of β2-AR KO mice showed reduction of β2-AR expression in the hippocampus and mPFC, which are involved in cognitive function, emotional regulation, self-regulation, goal-directed behaviors, neuroendocrine and autonomic function (McEwen and Morrison, 2013). It is reported that β2-AR activation in mPFC promotes memory consolidation through cAMP-PKA signaling (Zhou et al., 2013) and the expression of β2-AR in hippocampal CA3 is required for long-term memory consolidation (Zheng et al., 2015). However, the roles of β2-AR expression in the regulation of emotional behaviors are largely unknown.

In this study, β2-AR KO and HET mice showed decreased duration in the light compartment and open arms in L/D box and EPM tasks, indicating an increased anxiety or innate fear by β2-AR deletion. Considering that β1-AR antagonism attenuates the anxiety-like behavior during early withdrawal from chronic cocaine administration in rats, and β1-AR antagonist infusion in the basolateral amygdala facilitated anxiolytic effect (Rudoy and Van Bockstaele, 2007; Fu et al., 2008). Our data suggest that the balance between β1-AR and β2-AR might be critical for the regulation sensitivity of anxiety-like behavior. However, it is also possible that β2-AR KO might influence the cardiovascular system, which also correlates with the anxiety level (Nabi et al., 2010; Roest et al., 2010; Balasubramaniyan et al., 2016; Williams et al., 2016). In this study, no difference in the exploration of central area in OF task was detected, not consistent with the results of reduced anxiety in L/D box and EPM test. This discrepancy may be ascribed to the size of box applied for OF task. The box used for OF was relatively small (20 × 20 cm^2^) and not sensitive for examining the innate anxiety level in mice.

Considering the high rate of comorbid anxiety and depression disorders (Aderka et al., 2015), depression level of mice was subsequently tested by TST and FST, the classic animal models for detecting depression-like behavior (Cryan and Mombereau, 2004; Cryan et al., 2005; Petit-Demouliere et al., 2005). In this study, β2-AR KO mice exhibited an antidepressant-like behavior, characterized by decreased immobility time in the TST, but not in FST task.

β-ARs also play a critical role in drug addiction. β-AR blockade by propranolol produces a dose-related increase in cocaine-induced motor activity and a decrease in cocaine self-administration (Harris et al., 1996); impairs the retrieval, expression and reinstatement of cocaine-CPP in rats (Otis and Mueller, 2011; Otis et al., 2014); disrupts alcohol withdrawal in human and naloxone-precipitated morphine withdrawal in rats (Carlsson and Fasth, 1976; Bouton, 1993; Watanabe et al., 2003). However, the effects of β2-AR in drug addiction have not been identified yet. In our study, β2-AR KO mice showed increased response and higher rewarding properties to cocaine in the locomotion and CPP tests. Additionally, β2-AR KO mice showed high motivational effects of cocaine in coincident with the higher anxiety-like behavior observed in elevated plus maze and light-dark box model, consistent with the previous studies, which demonstrated that anxiety-like behavior predicted cocaine-CPP (i.e., the more anxious mice showed more preference for the cocaine-paired compartment) and anxiety increased vulnerability for cocaine (Ladrón de Guevara-Miranda et al., 2016). Hence, β2-AR may be a potential target for the treatment of anxiety and cocaine addiction.

In another aspect, the individual roles of β1/β2-AR in cocaine addiction are not clear. Vranjkovic et al. (2012) reported that both WT and β1/β2-AR double KO mice developed CPP, and no difference in cocaine-CPP was detected between both genotypes. Intriguingly, we also found that β1-AR antagonist betaxolol injected before cocaine conditioning did not completely attenuate the preference for cocaine in β2-AR KO mice, but only reduced the CPP scores to a similar level of WT mice after cocaine-CPP training. Accordingly, we speculated that β1-AR and β2-AR might play opposite roles in the regulation of cocaine CPP. Previous studies also suggested that selective blockade of β1-AR not β2-AR induces a persistent retrieval deficit of cocaine-associated memory and contextual fear conditioning (Murchison et al., 2004; Fitzgerald et al., 2016). Furthermore, both β1-AR and β2-AR can influence memory reconsolidation (Bernardi et al., 2009; Liu et al., 2015). However, the role of different types of β-AR in drug addiction and psychiatric disorders needs further research by β2-AR antagonist or β1-AR agonist. For example, antagonism of β2-AR in the β1-AR knockout mice or activation of β1-AR in the WT mice will be performed next to make it more clearly how these two β-ARs affect cocaine-CPP. Moreover, conditional knockout of β1-AR or β2-AR will be used to find out the role of β-ARs in different brain regions in anxiety, depression or rewarding properties of cocaine.

Taken together, our study indicates that β2-AR play critical roles in regulating anxiety-like behaviors and rewarding properties of cocaine. Our findings provide new insights into the physiological and pathological roles of β2-AR and a potential target for treatment of anxiety disorder and cocaine abuse.

## Author Contributions

XL and LM designed the research. XL analyzed the data and wrote the article. HZ, ZL and YZ performed the research and analyzed the data. XY and BX performed the research.

## Funding

This research was supported by grants from the National Natural Science Foundation of China (31430033, 91632307, 31571036, 31421091, 31371136), and Ministry of Science and Technology (2015CB553501, 2013CB835102, 2014CB942801).

## Conflict of Interest Statement

The authors declare that the research was conducted in the absence of any commercial or financial relationships that could be construed as a potential conflict of interest.

## References

[B1] Aderka I. M. Beard C. Lee J. Weiss R. B. Björgvinsson T. (2015). The relationship between depression and generalized anxiety during intensive psychological and pharmacological treatment. J. Affect. Disord. 184, 261–268. 10.1016/j.jad.2015.05.054 26118754

[B2] Al-Hasani R. McCall J. G. Foshage A. M. Bruchas M. R. (2013). Locus coeruleus kappa-opioid receptors modulate reinstatement of cocaine place preference through a noradrenergic mechanism. Neuropsychopharmacology 38, 2484–2497. 10.1038/npp.2013.151 23787819 PMC3799068

[B3] Asanuma M. Ogawa N. Mizukawa K. Haba K. Hirata H. Mori A. (1991). Distribution of the beta-2 adrenergic receptor messenger RNA in the rat brain by in situ hybridization histochemistry: effects of chronic reserpine treatment. Neurochem. Res. 16, 1253–1256. 10.1007/bf00966654 1664493

[B4] Balasubramaniyan N. Rayapati D. K. Puttiah R. H. Tavane P. Singh S. E. Rangan V.. (2016). Evaluation of anxiety induced cardiovascular response in known hypertensive patients undergoing exodontia—a prospective study. J. Clin. Diagn. Res. 10, ZC123–ZC127. 10.7860/JCDR/2016/19685.8391 27656554 PMC5028492

[B5] Bernardi R. E. Ryabinin A. E. Berger S. P. Lattal K. M. (2009). Post-retrieval disruption of a cocaine conditioned place preference by systemic and intrabasolateral amygdala β2- and α1-adrenergic antagonists. Learn. Mem. 16, 777–789. 10.1101/lm.1648509 19940038 PMC2788211

[B6] Birkett M. A. Shinday N. M. Kessler E. J. Meyer J. S. Ritchie S. Rowlett J. K. (2011). Acute anxiogenic-like effects of selective serotonin reuptake inhibitors are attenuated by the benzodiazepine diazepam in BALB/c mice. Pharmacol. Biochem. Behav. 98, 544–551. 10.1016/j.pbb.2011.03.006 21397628 PMC3085089

[B7] Blanchard R. J. McKittrick C. R. Blanchard D. C. (2001). Animal models of social stress: effects on behavior and brain neurochemical systems. Physiol. Behav. 73, 261–271. 10.1016/s0031-9384(01)00449-8 11438351

[B8] Bluett R. J. Gamble-George J. C. Hermanson D. J. Hartley N. D. Marnett L. J. Patel S. (2014). Central anandamide deficiency predicts stress-induced anxiety: behavioral reversal through endocannabinoid augmentation. Transl. Psychiatry 4:e408. 10.1038/tp.2014.53 25004388 PMC4119220

[B9] Bouton M. E. (1993). Context, time, and memory retrieval in the interference paradigms of Pavlovian learning. Psychol. Bull. 114, 80–99. 10.1037//0033-2909.114.1.80 8346330

[B10] Cahill L. Alkire M. T. (2003). Epinephrine enhancement of human memory consolidation: interaction with arousal at encoding. Neurobiol. Learn. Mem. 79, 194–198. 10.1016/s1074-7427(02)00036-9 12591227

[B11] Cahill L. Prins B. Weber M. McGaugh J. L. (1994). β-adrenergic activation and memory for emotional events. Nature 371, 702–704. 10.1038/371702a0 7935815

[B12] Carlsson C. Fasth B. G. (1976). A comparison of the effects of propranolol and diazepam in alcoholics. Br. J. Addict. Alcohol Other Drugs 71, 321–326. 10.1111/j.1360-0443.1976.tb00102.x 795446

[B101] Cronk J. C. Derecki N. C. Ji E. Xu Y. Lampano A. E. Smirnov I.. (2015). Methyl-CpG binding protein 2 regulates microglia and macrophage gene expression in response to inflammatory stimuli. Immunity 42, 679–691. 10.1016/j.immuni.2015.03.013 25902482 PMC4407145

[B13] Cryan J. F. Mombereau C. (2004). In search of a depressed mouse: utility of models for studying depression-related behavior in genetically modified mice. Mol. Psychiatry 9, 326–357. 10.1038/sj.mp.4001457 14743184

[B14] Cryan J. F. Valentino R. J. Lucki I. (2005). Assessing substrates underlying the behavioral effects of antidepressants using the modified rat forced swimming test. Neurosci. Biobehav. Rev. 29, 547–569. 10.1016/j.neubiorev.2005.03.008 15893822

[B15] De Paermentier F. Cheetham S. C. Crompton M. R. Katona C. L. Horton R. W. (1989). Lower cortical beta-adrenoceptor binding sites in post-mortem samples from depressed suicide victims. Br. J. Pharmacol. 98:818P. 2558766

[B16] Famularo R. Kinscherff R. Fenton T. (1988). Propranolol treatment for childhood posttraumatic stress disorder, acute type. A pilot study. Am. J. Dis. Child. 142, 1244–1247. 10.1001/archpedi.1988.02150110122036 3177336

[B17] Fitzgerald M. K. Otis J. M. Mueller D. (2016). Dissociation of β1- and β2-adrenergic receptor subtypes in the retrieval of cocaine-associated memory. Behav. Brain Res. 296, 94–99. 10.1016/j.bbr.2015.08.030 26318933 PMC4659763

[B18] Fu A. Li X. Zhao B. (2008). Role of β_1_-adrenoceptor in the basolateral amygdala of rats with anxiety-like behavior. Brain Res. 1211, 85–92. 10.1016/j.brainres.2008.03.013 18423428

[B19] Gao V. Suzuki A. Magistretti P. J. Lengacher S. Pollonini G. Steinman M. Q.. (2016). Astrocytic β2-adrenergic receptors mediate hippocampal long-term memory consolidation. Proc. Natl. Acad. Sci. U S A 113, 8526–8531. 10.1073/pnas.1605063113 27402767 PMC4968707

[B20] Green S. A. Holt B. D. Liggett S. B. (1992). β 1- and β 2-adrenergic receptors display subtype-selective coupling to Gs. Mol. Pharmacol. 41, 889–893. 1350321

[B102] Hara M. Takahashi T. Mitsumasu C. Igata S. Takano M. Minami T.. (2015). Disturbance of cardiac gene expression and cardiomyocyte structure predisposes *Mecp2*-null mice to arrhythmias. Sci. Rep. 5:11204. 10.1038/srep11204 26073556 PMC4466896

[B22] Harris G. C. Hedaya M. A. Pan W. J. Kalivas P. (1996). β-adrenergic antagonism alters the behavioral and neurochemical responses to cocaine. Neuropsychopharmacology 14, 195–204. 10.1016/0893-133x(95)00089-v 8866703

[B23] Heal D. J. Prow M. R. Buckett W. R. (1991). Effects of antidepressant drugs and electroconvulsive shock on pre- and postsynaptic α 2-adrenoceptor function in the brain: rapid down-regulation by sibutramine hydrochloride. Psychopharmacology (Berl) 103, 251–257. 10.1007/bf02244212 1851309

[B24] Kaster M. P. Budni J. Santos A. R. Rodrigues A. L. (2007). Pharmacological evidence for the involvement of the opioid system in the antidepressant-like effect of adenosine in the mouse forced swimming test. Eur. J. Pharmacol. 576, 91–98. 10.1016/j.ejphar.2007.08.026 17868670

[B25] Kessler R. C. Chiu W. T. Demler O. Merikangas K. R. Walters E. E. (2005). Prevalence, severity, and comorbidity of 12-month DSM-IV disorders in the national comorbidity survey replication. Arch. Gen. Psychiatry 62, 617–627. 10.1001/archpsyc.62.6.617 15939839 PMC2847357

[B103] Kishi N. Macklis J. D. (2004). MECP2 is progressively expressed in post-migratory neurons and is involved in neuronal maturation rather than cell fate decisions. Mol. Cell. Neurosci. 27, 306–321. 10.1016/j.mcn.2004.07.006 15519245

[B26] Ladrón de Guevara-Miranda D. Pavón F. J. Serrano A. Rivera P. Estivill-Torrus G. Suarez J.. (2016). Cocaine-conditioned place preference is predicted by previous anxiety-like behavior and is related to an increased number of neurons in the basolateral amygdala. Behav. Brain Res. 298, 35–43. 10.1016/j.bbr.2015.10.048 26523857

[B27] Lehmann M. L. Herkenham M. (2011). Environmental enrichment confers stress resiliency to social defeat through an infralimbic cortex-dependent neuroanatomical pathway. J. Neurosci. 31, 6159–6173. 10.1523/JNEUROSCI.0577-11.2011 21508240 PMC3094574

[B28] Levy F. O. Zhu X. Kaumann A. J. Birnbaumer L. (1993). Efficacy of beta 1-adrenergic receptors is lower than that of beta 2-adrenergic receptors. Proc. Natl. Acad. Sci. U S A 90, 10798–10802. 10.1073/pnas.90.22.10798 8248173 PMC47865

[B29] Liu X. Ma L. Li H. H. Huang B. Li Y. X. Tao Y. Z.. (2015). β-Arrestin-biased signaling mediates memory reconsolidation. Proc. Natl. Acad. Sci. U S A 112, 4483–4488. 10.1073/pnas.1421758112 25831532 PMC4394255

[B104] Lombardi L. M. Baker S. A. Zoghbi H. Y. (2015). MECP2 disorders: from the clinic to mice and back. J. Clin. Invest. 125, 2914–2923. 10.1172/JCI78167 26237041 PMC4563741

[B31] McEwen B. S. Morrison J. H. (2013). The brain on stress: vulnerability and plasticity of the prefrontal cortex over the life course. Neuron 79, 16–29. 10.1016/j.neuron.2013.06.028 23849196 PMC3753223

[B32] McGaugh J. L. Introini-Collison I. B. (1987). Hormonal and neurotransmitter interactions in the modulation of memory storage: involvement of the amygdala. Int. J. Neurol. 21–22, 58–72. 2908791

[B33] McReynolds J. R. Vranjkovic O. Thao M. Baker D. A. Makky K. Lim Y.. (2014). Beta-2 adrenergic receptors mediate stress-evoked reinstatement of cocaine-induced conditioned place preference and increases in CRF mRNA in the bed nucleus of the stria terminalis in mice. Psychopharmacology (Berl) 231, 3953–3963. 10.1007/s00213-014-3535-0 24696080 PMC8647032

[B34] Mellios N. Woodson J. Garcia R. I. Crawford B. Sharma J. Sheridan S. D.. (2014). β2-Adrenergic receptor agonist ameliorates phenotypes and corrects microRNA-mediated IGF1 deficits in a mouse model of Rett syndrome. Proc. Natl. Acad. Sci. U S A 111, 9947–9952. 10.1073/pnas.1309426111 24958851 PMC4103343

[B36] Mufaddel A. Osman O. T. Almugaddam F. Jafferany M. (2013). A review of body dysmorphic disorder and its presentation in different clinical settings. Prim Care Companion CNS Disord. 15:PCC.12r01464. 10.4088/PCC.12r01464 24392251 PMC3869603

[B38] Murchison C. F. Zhang X. Y. Zhang W. P. Ouyang M. Lee A. Thomas S. A. (2004). A distinct role for norepinephrine in memory retrieval. Cell 117, 131–143. 10.1016/s0092-8674(04)00259-4 15066288

[B39] Nabi H. Hall M. Koskenvuo M. Singh-Manoux A. Oksanen T. Suominen S.. (2010). Psychological and somatic symptoms of anxiety and risk of coronary heart disease: the health and social support prospective cohort study. Biol. Psychiatry 67, 378–385. 10.1016/j.biopsych.2009.07.040 19819425 PMC2963017

[B40] Ordway G. A. Gambarana C. Frazer A. (1988). Quantitative autoradiography of central beta adrenoceptor subtypes: comparison of the effects of chronic treatment with desipramine or centrally administered l-isoproterenol. J. Pharmacol. Exp. Ther. 247, 379–389. 10.1161/01.res.62.1.173 2845059

[B42] Otis J. M. Fitzgerald M. K. Mueller D. (2014). Inhibition of hippocampal β-adrenergic receptors impairs retrieval but not reconsolidation of cocaine-associated memory and prevents subsequent reinstatement. Neuropsychopharmacology 39, 303–310. 10.1038/npp.2013.187 23907403 PMC3870790

[B41] Otis J. M. Mueller D. (2011). Inhibition of β-adrenergic receptors induces a persistent deficit in retrieval of a cocaine-associated memory providing protection against reinstatement. Neuropsychopharmacology 36, 1912–1920. 10.1038/npp.2011.77 21544069 PMC3154110

[B43] Petit-Demouliere B. Chenu F. Bourin M. (2005). Forced swimming test in mice: a review of antidepressant activity. Psychopharmacology (Berl) 177, 245–255. 10.1007/s00213-004-2048-7 15609067

[B44] Porsolt R. D. Bertin A. Jalfre M. (1977). Behavioral despair in mice: a primary screening test for antidepressants. Arch. Int. Pharmacodyn. Ther. 229, 327–336. 596982

[B45] Pravetoni M. Wickman K. (2008). Behavioral characterization of mice lacking GIRK/Kir3 channel subunits. Genes Brain Behav. 7, 523–531. 10.1111/j.1601-183x.2008.00388.x 18194467

[B105] Rehsia N. S. Dhalla N. S. (2010). Mechanisms of the beneficial effects of beta-adrenoceptor antagonists in congestive heart failure. Exp. Clin. Cardiol. 15, e86–e95. 21264074 PMC3016066

[B46] Roest A. M. Martens E. J. de Jonge P. Denollet J. (2010). Anxiety and risk of incident coronary heart disease: a meta-analysis. J. Am. Coll. Cardiol. 56, 38–46. 10.1016/j.jacc.2010.03.034 20620715

[B47] Rudoy C. A. Van Bockstaele E. J. (2007). Betaxolol, a selective β_1_-adrenergic receptor antagonist, diminishes anxiety-like behavior during early withdrawal from chronic cocaine administration in rats. Prog. Neuropsychopharmacol. Biol. Psychiatry 31, 1119–1129. 10.1016/j.pnpbp.2007.04.005 17513029 PMC4287233

[B48] Shonesy B. C. Bluett R. J. Ramikie T. S. Báldi R. Hermanson D. J. Kingsley P. J.. (2014). Genetic disruption of 2-arachidonoylglycerol synthesis reveals a key role for endocannabinoid signaling in anxiety modulation. Cell Rep. 9, 1644–1653. 10.1016/j.celrep.2014.11.001 25466252 PMC4268380

[B49] Steenen S. A. van Wijk A. J. van der Heijden G. J. van Westrhenen R. de Lange J. de Jongh A. (2016). Propranolol for the treatment of anxiety disorders: systematic review and meta-analysis. J. Psychopharmacol. 30, 128–139. 10.1177/0269881115612236 26487439 PMC4724794

[B50] Steru L. Chermat R. Thierry B. Simon P. (1985). The tail suspension test: a new method for screening antidepressants in mice. Psychopharmacology (Berl) 85, 367–370. 10.1007/bf00428203 3923523

[B106] Tai D. J. Liu Y. C. Hsu W. L. Ma Y. L. Cheng S. J. Liu S. Y.. (2016). MeCP2 SUMOylation rescues Mecp2-mutant-induced behavioural deficits in a mouse model of Rett syndrome. Nat. Commun. 7:10552. 10.1038/ncomms10552 26842955 PMC4743023

[B51] Vranjkovic O. Hang S. Baker D. A. Mantsch J. R. (2012). β-adrenergic receptor mediation of stress-induced reinstatement of extinguished cocaine-induced conditioned place preference in mice: roles for β1 and β2 adrenergic receptors. J. Pharmacol. Exp. Ther. 342, 541–551. 10.1124/jpet.112.193615 22593095 PMC3400797

[B53] Watanabe T. Nakagawa T. Yamamoto R. Maeda A. Minami M. Satoh M. (2003). Involvement of noradrenergic system within the central nucleus of the amygdala in naloxone-precipitated morphine withdrawal-induced conditioned place aversion in rats. Psychopharmacology (Berl) 170, 80–88. 10.1007/s00213-003-1504-0 12768272

[B55] Williams S. E. Carroll D. Veldhuijzen van Zanten J. J. Ginty A. T. (2016). Anxiety symptom interpretation: a potential mechanism explaining the cardiorespiratory fitness-anxiety relationship. J. Affect. Disord. 193, 151–156. 10.1016/j.jad.2015.12.051 26773908

[B56] Wohleb E. S. Hanke M. L. Corona A. W. Powell N. D. Stiner L. M. Bailey M. T.. (2011). β-Adrenergic receptor antagonism prevents anxiety-like behavior and microglial reactivity induced by repeated social defeat. J. Neurosci. 31, 6277–6288. 10.1523/JNEUROSCI.0450-11.2011 21525267 PMC3160240

[B57] Zheng J. Luo F. Guo N. N. Cheng Z. Y. Li B. M. (2015). β1-and β2-adrenoceptors in hippocampal CA3 region are required for long-term memory consolidation in rats. Brain Res. 1627, 109–118. 10.1016/j.brainres.2015.08.035 26343545

[B58] Zhou H. C. Sun Y. Y. Cai W. He X. T. Yi F. Li B. M.. (2013). Activation of β2-adrenoceptor enhances synaptic potentiation and behavioral memory via cAMP-PKA signaling in the medial prefrontal cortex of rats. Learn. Mem. 20, 274–284. 10.1101/lm.030411.113 23596314

